# A rare case of Brown-Sequard syndrome caused by cervical disc herniation: a case report

**Published:** 2012-11

**Authors:** Amir Abbas Ghasemi

**Affiliations:** ^a^Department of Neurosurgery, Urmia University of Medical Sciences,Urmia, Iran.

## Abstract

Brown-Sequard syndrome is a rare neurological disorder characterized by ipsilateral motor paralysis caused by a lesion through corticospinal tract and contralateral loss of pain and temperature sensation due to the involvement of spinothalamic tract. Cervical disc herniation has been reported to be a rare cause of Brown-Séquard's syndrome. This paper aims to report a case of Brown-Sequard syndrome that occurred in a patient suffering from CHD.

In this case, using a rapid and urgent intervention we could prevent permanent neurologic deficit in the patient.

**Case:**

A 56-year-old woman complained about a sudden paresis in her right leg lasting for 4 days. Her pain was progressively worsening until she couldn’t walk without assistance. There was no history of trauma in the neck. Neurological examinations revealed right side spastic hemi-paresis as well as loss of pain and temperature sensation below T4 dermatome in the left side. The case was diagnosed as Brown-Sequard syndrome and cervical magnetic resonance imaging scan showed a disc herniation at C5/C6 and C6/C7 levels. Surgery was performed via anterior cervical microdiscectomy and fusion. After a 2-month period of follow-up, neurological assessments showed that motor and sensory functions of the patient returned to the normal condition.

Although cervical disc herniation as a cause of Brown-Sequard syndrome is relatively rare, early diagnosis accompanied by an urgent treatment can prevent neurological complications in such cases.

**Keywords:**

Brown-Sequard Syndrome, Cervical Disc Herniation, Surgery, Ipsilateral motor paralysis


					Volume 4, Suppl. 1 Nov 2012
Publisher: Kermanshah University of Medical Sciences
URL: http://www.jivresearch.org
Copyright:
Congress of Iranian Neurosurgeons, 14-16 November 2012, Kermanshah, Iran
Paper No. 82
A rare case of Brown-Sequard syndrome caused by cervical
disc herniation: a case report
Amir Abbas Ghasemi a,*
a
Department of Neurosurgery, Urmia University of Medical Sciences, Urmia, Iran.
Abstract:
Brown-Sequard syndrome is a rare neurological disorder characterized by ipsilateral motor paralysis caused by a
lesion through corticospinal tract and contralateral loss of pain and temperature sensation due to the involvement of
spinothalamic tract. Cervical disc herniation has been reported to be a rare cause of Brown-Sequard's syndrome. This
paper aims to report a case of Brown-Sequard syndrome that occurred in a patient suffering from CHD.
In this case, using a rapid and urgent intervention we could prevent permanent neurologic deficit in the patient.
Case: A 56-year-old woman complained about a sudden paresis in her right leg lasting for 4 days. Her pain was
progressively worsening until she couldn't walk without assistance. There was no history of trauma in the neck.
Neurological examinations revealed right side spastic hemi-paresis as well as loss of pain and temperature sensation
below T4 dermatome in the left side. The case was diagnosed as Brown-Sequard syndrome and cervical magnetic
resonance imaging scan showed a disc herniation at C5/C6 and C6/C7 levels. Surgery was performed via anterior
cervical microdiscectomy and fusion. After a 2-month period of follow-up, neurological assessments showed that
motor and sensory functions of the patient returned to the normal condition.
Although cervical disc herniation as a cause of Brown-Sequard syndrome is relatively rare, early diagnosis
accompanied by an urgent treatment can prevent neurological complications in such cases.
Keywords:
Brown-Sequard Syndrome, Cervical Disc Herniation, Surgery, Ipsilateral motor paralysis
* Corresponding Author at:
Amir Abbas Ghasemi: Department of Neurosurgery, Urmia University of Medical Sciences, Urmia, Iran, Tel: 09122038271,
Email: dr.amirghasemi@ymail.com, (Ghasemi AA.).

				

